# Epidemiology of Viral Hepatitis B and C Infections in Ibb City, Yemen

**DOI:** 10.5812/hepatmon.6140

**Published:** 2012-07-30

**Authors:** Rajesh Nivarti Gacche, Al Mohani Sadiq Kaid

**Affiliations:** 1 Biotechnology department, School of Life Sciences, Swami Ramanand Teerth Marathwada University, Nanded, India

**Keywords:** Hepatitis B virus, Hepacivirus, Epidemiology, Yemen

## Abstract

**Background:**

The global epidemic of hepatitis B and hepatitis C is a serious publichealth problem. Chronic hepatitis B and hepatitis C are among the leading causes of preventable death worldwide. World Health Organization (WHO) estimates that up to two billion people in the world have been infected with HBV; about 350 million people live with chronic HBV infection, and about 600,000 people die from HBV- related liver disease or HCC each year. The endemicity of infection is considered high in Yemen. Data for prevalence of HBsAg and HCV antibodies in Ibb city in Yemen is rare and inadequate.

**Objectives:**

The study was undertaken to study the epidemiology and prevalence of viral hepatitis (HBV) and (HCV) in Ibb city, Yemen.

**Patients and Methods:**

554 pre-designed questionnaires and sera samples were collected in July 2010. Sera were tested for HBsAg and HCV antibodies by ELISA quantitative technique. Each individual’s data were collected in a pre-designed questionnaire.

**Results:**

The prevalence of HBV in Ibb city was 1.81 %, whereas, the prevalence of HCV was 1.99 %.

**Conclusions:**

This study revealed low level risk of hepatitis B virus and hepatitis C virus infections. Inadequate information on the prevalence and risk determinants of viral hepatitis among the different population groups in Yemen are responsible about morbidity and mortality of HBV and HCV in Ibb city, Yemen.

## 1. Background

The global epidemic of hepatitis B and hepatitis C is a serious public- health problem. Chronic hepatitis B and hepatitis C are among the leading causes of preventable death worldwide [1]. It is estimated that HBV and HCV infections cause nearly a million death cases each year. The World Health Organization (WHO) estimates that up to two billion people in the world have been infected with HBV; about 350 million people live with chronic HBV infection, and about 600,000 people die from HBV- related liver disease or HCC each year [2][3]. The prevalence of chronic HBV infection varies geographically, from high (> 8 %), intermediate (2-7 %) to low (< 2 %) prevalence [4]. The endemicity of infection is considered high in Yemen, where prevalence of positive HBsAg ranges from 8 % to 20 %, and up to 50 % of the populations generally have serological evidence of previous HBV infection [5]. In other studies, the prevalence of HBsAg in Yemen is 12.7 % –18.5 % [6]. However, the prevalence of antibodies to HCV is 1.7 % in healthy volunteers [7]. Most of epidemiological studies were done in different cities in Yemen, the prevalence rates of HBsAg and HCV antibodies are 10.5 % and 2.3 % in Sana’a, 4.75 % and 0.6 % in Aden, 5.6 % and 0.8 % in Hajah, 26.3 % and 5.1 % in Soqotra respectively [8]. Although Ibb city is the densest populated governorate outside of Sana’a city and it has the largest Yemeni expatriates abroad, data for prevalence of HBsAg and HCV antibodies in Ibb city were rare and inadequate.

## 2. Objectives

The main aim of this study was to determine the prevalence of HBsAg and HCV antibodies among population in Ibb city, and to determine the risk factors using HBsAg and HCV antibodies as indicators for infection.

## 3. Patients and Methods

This work is carried out within the epidemiological and laboratory field work in July 2010. 554 specimens were randomly selected by systematic random sampling of every 5th house in Ibb city. A full history was taken from each studied individual and the findings recorded in a pre-designed questionnaire. Four milliliters of whole blood were collected from each subject. Then the sera were separated. HBsAg and HCV antibodies were detected by commercial kits (One step HBsAg Test, Intec, China) and (Rapid Anti-HCV Test, Intec, China). Positive samples were confirmed by enzyme immunoassay (EIA) for hepatitis B surface antigen and HCV antibodies with commercially kits (DRG, HBsAg, USA) and (DRG, HCV antibodies, USA). All collected data were analyzed using SPSS program.

## 4. Results

**Figure 1:**
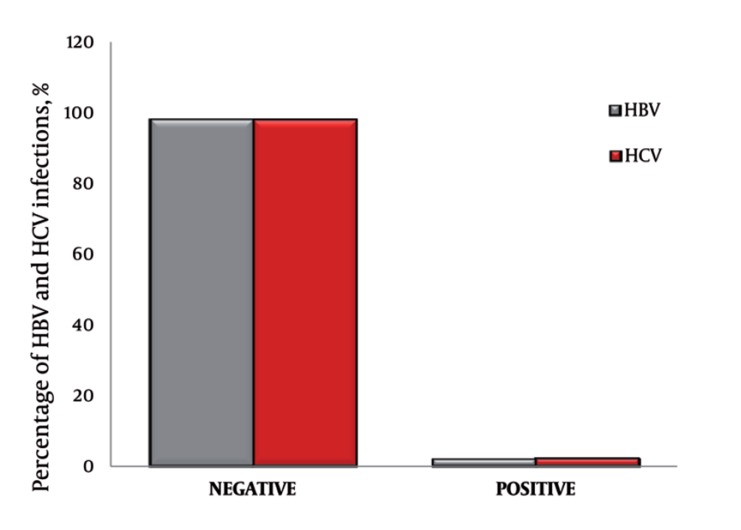
Shows Percentage of HBV and HCV Infections in Ibb City

A total of 554 volunteers completed the study questionnaire and donated blood. 69.7 % (386) were females. Figure 1 outlines prevalence of HBV and HCV in Ibb city; the percentages were 1.81 % (10) and 1.99 % (11) respectively. The percentage of HBV and HCV infections among females were 1.62 % (9) and 1.81 % (10) respectively, which is higher than in males. Individuals who aged between 55 – 62 years old had the highest percentage of HBV and HCV infections, which were 0.54% (3) and 0.72% (4) respectively as illustrated in Table 1. About 74 % (410) of tested people had an idea about viral hepatitis and the sources of their knowledge were in schools or collages which represented 52.2 % (289) of others sources. The percentages of HBV and HCV morbidity according to tested individuals among their family were 4 % (4) and 21 % (19) respectively, whereas, the percentage of HBV and HCV morbidity among non-family members were 10 % (9) and 13 % (11) respectively. The mortality of viral hepatitis was 22 % (123). Out of this percentage, only 3 % (4) of death contributed to HBV infection and 18 % (22) to HCV infection.

**Table 1:** Prevalence of Hepatitis B Virus and Hepatitis C Virus Infections Among Screened Participants by Selected Demographic Characteristics

	**Patients, No. (%)**	**HBsAg a Positive, No. (%)**	**P value**	**HCV ABs a, No. (%)**	**P value**
**Gender**			0.142		0.106
Male	168 (30.3)	1 (0.18)		1 (0.18)	
Female	386 (69.70	9 (1.62)		10 (1.81)	
**Age group, y**			0.000		0.000
≤ 14	54 (9.7)	00 (00)		00 (00)	
15-22	214 (38.6)	2 (0.36)		1 (0.18)	
23-30	143 (25.8)	1 (0.18)		2 (0.36)	
31-38	55 (9.9)	00 (00)		00 (00)	
39-46	32 (5.8)	1 (0.18)		1 (0.18)	
47-54	22 (4)	2 (0.36)		2 (0.36)	
55-62	20 (3.6)	3 (0.54)		4 (0.72)	
63-70	9 (1.6)	00 (00)		00 (00)	
≥ 71	5 (0.9)	1 (0.18)		1 (0.18)	
**Knowledge of viral hepatitis **			0.240		0.306
Yes, I have knowledge	410 (74)	6 (1.09)		7 (1.27)	
No, I have no knowledge	142 (25.6)	4 (0.72)		4 (0.72)	
Missing value	2 (0.4)	-		-	
**Source of Knowledge of viral hepatitis**					
Friends and Relatives	89 (16.1)	00 (00)		1 (1.12)	
TV	45 (8.1)	00 (00)		00 (00)	
Radio	6 (1.1)	00 (00)		00 (00)	
Newspapers	76 (13.7)	1 (1.32)		1 (1.32)	
Hospital and Clinics	68 (12.3)	00 (00)		1 (1.47)	
Others sources	289 (52.2)	5 (1.73)		5 (1.73)	
**Knowledge of viral hepatitis infection**			0.066		0.076
Yes, I am infected	8 (1.4)	1 (0.18)		1 (0.18)	
No, I am not infected	261 (47.1)	5 (0.9)		6 (1.08)	
I do not know	284 (51.3)	4 (0.72)		4 (0.72)	
Missing value	1 (0.2)	-		-	

^a^ Abbreviations: HBsAg, hepatitis B surface antigene; HCV ABs, hepatitis C virus antibodies

## 5. Discussion

Prevalence of HBV and HCV may be different in different regions and various groups of the same community. Previous studies revealed prevalence rates of HBV as 10.5 % in Sana’a, 4.75 % in Aden, 5.6 % in Hajah, 26.3 % in Soqotra and 2.7 % in Mukala. This study estimated that the prevalence of HBV exposure in the population of Ibb city was 1.81 %. However, previous studies revealed prevalence rates of HCV as 2.37 % in Sana’a, 0.6% in Aden, 0.8 % in Hajah, and 5.1 % in Soqotra. This study estimated that the prevalence of HCV exposure in the population of Ibb city was 1.99 %. According to WHO, Yemen has an intermediate level, which is 2 % -7 % of HBV and 2.5 % - 4.9 % of HCV. The decline in prevalence rate of HBV in Ibb city may be attributed to many factors, such as increased HBV vaccination that Yemen introduced universal immunization against HBV for infants and high risk groups in early 2000, which contributed to combat hepatitis B virus. In addition, Ibb city has the largest Yemeni expatriates abroad distributed in Saudi Arabia; about 500,000 and USA; about 200,000, these countries insist that new comers should be free from viral hepatitis B and C. The percentages of HBV and HCV infections among females are higher than in males, this may be cause of females number in taken samples was higher. The highest HBV and HCV infections were 0.54 % and 0.72 % which observed in age group 55-62. These people had variable history of exposure to HBV and HCV risk factors such as major/dental surgery or blood transfusion. This increase could indicate an accumulated risk of infection over time. In addition, the results indicated that the horizontal spread of hepatitis B virus may be of a greater importance than vertical transmission. Our study is partially in agreement with previous studies. 52.2 % of tested subjects acquired their knowledge during their study in schools or collages. This indicates the weak role of health education programs in Yemen. The mortality of viral hepatitis was 22 %, from this percentage, only 3 % of death contributed to HBV infection and 18 % (22) to HCV infection. These results are similar to other studies. Inadequate information on the prevalence and risk determinants of viral hepatitis among the different population groups in Yemen are responsible about morbidity and mortality of HBV and HCV in Ibb city, Yemen.
